# Parkinson's Disease Polygenic Risk Score and Neurological Involvement in Carriers of the *FMR1* Premutation Allele: A Case for Genetic Modifier

**DOI:** 10.1002/mgg3.70043

**Published:** 2024-11-26

**Authors:** Danuta Z. Loesch, Freddy Chafota, Minh Q. Bui, Elsdon Storey, Anna Atkinson, Nicholas G. Martin, Scott D. Gordon, Miguel E. Rentería, Randi J. Hagerman, Flora Tassone

**Affiliations:** ^1^ School of Psychology and Public Health La Trobe University Bundoora Victoria Australia; ^2^ Mental Health & Neuroscience Program QIMR Berghofer Medical Research Institute Brisbane Queensland Australia; ^3^ Centre for Epidemiology and Biostatistics, School of Global and Population Health University of Melbourne Victoria Australia; ^4^ Department of Medicine (Neuroscience) Monash University, Alfred Hospital Campus Melbourne Australia; ^5^ Department of Pediatrics University of California Davis Health Sacramento California USA; ^6^ Medical Investigation of Neurodevelopmental Disorders (MIND) Institute University of California Davis Health Sacramento California USA; ^7^ Department of Biochemistry and Molecular Medicine University of California Davis California USA; ^8^ School of Medicine and MIND Institute University of California Davis Medical Center Davis California USA

**Keywords:** *FMR1*/CGG repeats, fragile X premutation, FXTAS, neurological status, Parkinson's risk score, premutation carriers, regression analysis, whole genome screening

## Abstract

**Background:**

Premutation alleles of the *FMR1* X‐linked gene containing CGG repeat expansions ranging from 55 to 200 are associated with diverse late‐onset neurological involvements, including most severe disorder termed Fragile X‐associated Tremor/Ataxia Syndrome (FXTAS). It is intriguing that at least one‐third of male, and a much lower than predicted from the X‐linkage proportion of female carriers are free of this syndrome. This suggests the existence of secondary genetic factors modifying the risk of neurological involvements in these carriers. Considering the occasional presence of parkinsonian features in FXTAS, we explored the possibility that the Parkinson's Disease Polygenic Risk Score (PD PRS) is related to the occurrence of FXTAS or less severe neurological involvements, in premutation carriers.

**Methods:**

The Genome‐wide SNP genotyping and clinical data on neurological status were obtained from 250 unrelated affected and non‐affected male and female adult carriers of the premutation. The medians for the Parkinson’s Disease Polygenic Risk Score (PD PRS) were compared between the groups of asymptomatic and neurologically affected carriers, and the association of PD PRS with neurological involvement in context with the other known risk factors was explored by fitting univariate and multiple logistic regression models.

**Results:**

There was a significant difference between the medians from the asymptomatic versus neurologically affected (FXTAS+) groups (*p* = 0.009). The FXTAS+ status was significantly associated with age at testing (*p* < 0.001), gender (*p* = 0.026), and with PD PRS (*p* = 0.021). The contribution of PD PRS remained significant after adjusting for age and gender (*p* = 0.044).

**Conclusions:**

We have obtained the first evidence for the relationship between PD PRS and the risk of FXTAS or lesser neurological involvements in the FMR1 premutation carriers. This suggests the role of Parkinson's disease polygenic variants as genetic modifiers of the risk of late onset neurological changes in these carriers.

## Graphical abstract

We have obtained the first evidence for the relationship between Parkinson's Disease Polygenic Risk Score and the risk of Fragile X‐associated Tremor/Ataxia Syndrome (FXTAS) or lesser neurological involvements in the FMR1 premutation carriers, independent of the causal effect of CGG allele repeat size. This suggests the role of Parkinson's disease polygenic variants as genetic modifier of the risk of late onset neurological changes in these carriers. Notably, these variants do not significantly contribute to the risk of parkinsonian features in premutation carriers affected with the constellation of FXTAS‐like neurological changes.

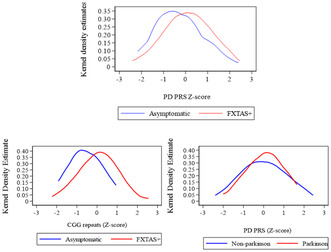

## 1 Introduction

Premutation in the *FMR1* (Fragile X messenger ribonucleoprotein 1) gene, representing the alleles with small to moderate expansions of CGG repeats in its non‐coding region, has been associated with a constellation of clinical conditions (Tassone et al. 2023). Fragile X‐associated Tremor/Ataxia Syndrome (FXTAS), a late onset neurodegenerative disorder, affecting up to 75% of males carrying this allele as they get older (Hagerman et al. 2001; Jacquemont et al. 2004) is the most severe. Since *FMR1* is an X‐linked gene, this syndrome is much less prevalent (approx. 16%) in female carriers due, in part, to the protective effect of the second (normal) X chromosome. This syndrome presents as gait ataxia, kinetic tremor, executive dysfunction, and parkinsonism in a proportion of the affected individuals. (Hagerman and Hagerman 2004). Typical changes in the brain consist of the white matter degeneration, predominantly in the upper cerebellar peduncles seen on MR images of the majority of male patients (MCP sign) (Brunberg et al. 2002), and the widespread presence of intranuclear inclusions in neurons and astroglia (Greco et al. 2002). However, in FXTAS females, the MCP sign is rare, since degeneration occurs more commonly in the splenium of the corpus callosum, which is recognized, according to the latest criteria of FXTAS, as minor diagnostic feature (Hall et al. 2017). The pathological mechanisms behind neurodegenerative changes have been recognized as sequestration of a number of proteins essential for brain metabolism caused by the elevation of the *FMR1*mRNA, on the one hand, and intracellular deposits of RAN translated FMRpolyG and FMRpolyA aggregates, on the other (Tassone et al. 2023). The latest comprehensive review of this syndrome has been given in: Cabal‐Herrera et al. (2020).

Since the original description of FXTAS in 2001, several definitions of this syndrome based on the diagnostic criteria have been introduced (Hall et al. 2014). “Definite” FXTAS, representing the most severe form, requires notable presence of one clinical and one radiological characteristic (major) features represented by MCP; “probable” FXTAS requires two clinical major, or one clinical major and one radiological minor change, represented by white matter degeneration in the corpus callosum; “possible” FXTAS represents uncertain diagnosis, where only two clinical features (one major and one minor) are present. Consequently, carrier females usually fall into the two latter categories (probable or possible) because of the usual absence of MCP sign. It has been shown that the risk of FXTAS and the age of onset correlates with increased CGG repeat numbers, with the highest number of observed cases risk falling between 80 and100 repeats (Tassone et al. 2023).

More recently, attention has been drawn to the existence of low‐symptomatic/mono‐symptomatic forms of neural involvement, especially in female premutation carriers. One, most relevant example are isolated tremors (Chonchaiya et al. 2009; Coffey et al. 2008; Fay‐Karmon and Hassin‐Baer 2019). It has also been shown in a female cohort assessed over the period of 10 years that the kinetic tremor rarely progress to a diagnosable FXTAS with aging (Loesch, Duffy, et al. 2021). Other types of occasionally reported neural involvements, including own observations, include isolated imbalance, dementia, or fibromyalgia.

Considering a diversity in the type or severity of clinical manifestations, combined with the fact that at least one‐third of carriers of this premutation remain symptom‐free, there is a possibility of the existence of secondary genetic factors that modify the risk of FXTAS, or any other fragile X associated neural involvement, in carriers of premutation allele. We have selected genetic variants encompassed by the Parkinson's Disease Polygenic Risk Score (PD PRS) as the likely candidate, considering the presence of parkinsonism in some individuals affected with this syndrome. This is still not sufficient evidence for a causal link between these two conditions, especially as screening for the *FMR1* premutation in cohorts of idiopathic PD (iPD) failed to show that these alleles account for a significant proportion of these cases (Toft et al. 2005; Hall et al. 2006; Kraff et al. 2007). On the other hand, cases with parkinsonism have been reported in premutation carriers who either did not show features diagnosable of FXTAS (Louis et al. 2006; Hall et al. 2009), or manifested both FXTAS and parkinsonian phenotypes (Jacquemont et al. 2004; Hall et al. 2009; Loesch et al. 2005). Given inconsistency of clinical results and the need for studies to help establish the real nature of the apparent association between these two phenotypes, in this study we explore the possibility that PD PRS is related to the occurrence and severity of FXTAS, or lesser neurological involvement, using a somewhat modified classification of these involvements. We present the first evidence for a significant effect of this score in the occurrence of FXTAS or lesser neurological involvement and discuss a possible underlying cause of this effect.

## 2 Material and Methods

### 2.1 Ethical Compliance

The Australian studies were approved by the La Trobe University Human Research Ethics Committee (HEC01‐85 and HEC15‐058). The studies carried out at the University of California were in accordance with the Institutional Review Board at the University of California, Davis. All participants gave written informed consent before participating in the study, in line with the Declaration of Helsinki.

### 2.2 Sample

DNA samples from carriers of the *FMR1* premutation were collected from two different cohorts. Forty‐one Australian carriers from La Trobe University, Melbourne, were combined with 209 American carriers seen at the MIND Institute, at the University of California, Davis. The only inclusion criteria were a *FMR1* premutation carrier status and adult age (> 40), and no pre‐selection.

The Australian sample comprised adult male and female carriers aged between 42 and 80 years (mean = 62.3; SD = 8.7). Except for one East Asian (Chinese) male, all participants were white Caucasians. These participants were originally recruited via referrals through fragile X families from the Victorian Genetic Counselling Clinic of the Murdoch Children's Research Institute, or from one of several neurology clinics associated with the University of Melbourne and Monash University; the minority (some residing in the other states) were self‐referred by postings in the community through the Australian Fragile X Association.

The American cohort comprised 209 premutation carriers aged between 40 and 85 (Mean = 63.8, SD = 9.3) from the University of California Davis, USA. They were participants in research studies involving premutation carriers conducted at the MIND Institute, originally identified through cascade testing of relatives of FXS probands. The majority of participants were white Caucasians, and nearly one‐third were of Hispanic ethnicity.

### 2.3 Assessment of Neurological Status

Assessment of neurological status has been conducted, in both cohorts, by neurologists experienced in movement disorders, and the FXTAS diagnosis was based on the established criteria (listed in the Introduction). The subsets of both cohorts have been followed up longitudinally, with the results of the latest assessment considered in this study. In the Australian sample, the assessments, carried out over the past two decades jointly in all the participants by two experienced neurologists (DL and ES), also included obtaining the Unified Parkinson's disease rating scale (UPDRS)‐Motor scores (Fahn et al. 1987).

In the American sample, assessment of neurological status/FXTAS diagnosis was conducted by several different neurologists experienced in movement disorders. Thus, the unified rankings of neurological involvement in the combined sample adopted for this study relied on comparable criteria between the two sites. Additional neurological features considered in this study was PD status represented by binary 0 or 1 scores, for an absence or presence of parkinsonism, respectively. In both cohorts, score 1 was based on the present of at least one the three diagnostic motor symptoms of PD, especially bradykinesia and/rigidity, regardless of the presence or absence of Fragile X‐associated neurological syndromic or non‐syndromic manifestations. In the Australian sample, a confirmatory criterion was the value of UPDRS score exceeding one standard deviation.

### 2.4 CGG Repeat Size

CGG repeat sizing to establish premutation status was conducted, in both cohorts, in the Laboratory of Dr. Tassone, at the MIND Institute, University of California Davis Medical Center, Sacramento, CA, USA. Genomic DNA was isolated from peripheral blood lymphocytes using standard methods (Puregene Kit; Gentra Inc. Minneapolis, MN). A combination of Southern blot and PCR analyses was used to determine methylation status and allele size as previously described (Tassone et al. 2008; Filipovic‐Sadic et al. 2010).

### 2.5 Genome‐Wide Array Genotyping

DNA samples were genotyped on the Illumina Global Screening Array version 3 at the Australian Translational Genomics Centre, Queensland University of Technology. Standard quality control measures were used before estimating the polygenic scores. We used genetic data from unrelated individuals to avoid biases due to population stratification and relatedness. We excluded all SNPs with a minor allele frequency < 1%; missingness rate > 5%; GenTrain score from GenomeStudio of < 0.6; or *p* < 1 × 10^−6^ in the Hardy–Weinberg Equilibrium chi‐squared test. Genotyped individuals were excluded if there was discordance between reported sex and that was determined by genotyping. Genetic imputation was performed through the Michigan Imputation Server, and we utilised the HRCr1.1 panel for reference.

We calculated PD PRS for each participant by standard procedure, as a linear combination of the participant's allele dosage across SNPs, weighted by the SNP effect size. Effect sizes were taken from the latest GWAS meta‐analysis of PD, which included 37,688 cases, 18,618 UK Biobank proxy‐cases, and 1.4 million controls (Nalls et al. 2019).

As expected, there was a correlation between effect sizes of adjacent genetic variants due to linkage disequilibrium (LD). To account for this, we used a powerful new method, SBayesR, to improve accuracy and reduce bias (Campos et al. 2021). This method performs Bayesian posterior inference by combining likelihood estimates of multiple regression coefficients with the PD GWAS Summary Statistics and a finite mixture of priors of normal distributions of the SNP effects (Zabad, Gravel, and Li 2023). SBayesR maximizes computational efficiency using the marginal GWAS summary statistics and the LD matrix sparsity (Lloyd‐Jones et al. 2019). 25,000 MCMC iterations were chosen for the chain length in SBayesR, with 5000 burn‐in iterations.

### 2.6 Statistical Analysis

**FIGURE 1:**
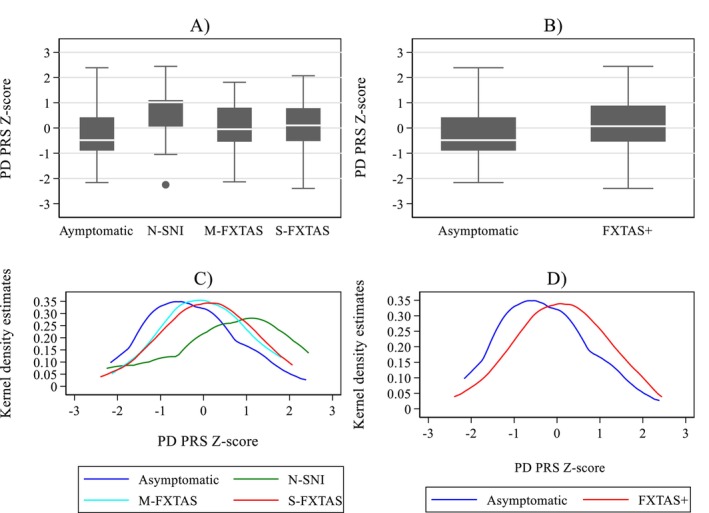
Distributions of the PD PRS *Z*‐score for four categories of neurological involvement: Asymptomatic, Non‐Syndromic (N‐SNI), Mild‐FXTAS (M‐FXTAS), and Severe‐FXTAS (S‐FXTAS) illustrated by boxplots (A) and Kernel density estimation plots (C); the corresponding distributions for the combined categories: Asymptomatic and FXTAS+, are in B and D, respectively.

Descriptive statistics are presented by means (*M*), standard deviations (SD), and proportions. In the present analysis, we considered four clinical categories ranked according to the severity of neurological involvement; alternatively, all three affected categories were summed up against the asymptomatic one (as detailed in Figure 1). Distributions of PD PRS across these four, and two categories, respectively, were illustrated by boxplots and Kernel density estimation plots. A nonparametric Mann–Whitney test was used for comparing the median PD PRS between two categories, and Kruskal–Wallis rank test‐ for comparing more than two categories. Two‐sample *t*‐test (for means), or *z*‐test (for proportions), were used to compare demographic characteristics between asymptomatic and FXTAS+ categories. Associations between FXTAS+ status as outcome, and age, sex, CGG repeats, and PD PRS as predictors were assessed through univariate and multiple logistic regression analyses.

Association between the presence or absence of parkinsonism categories and predictors (FXTAS+ and PD PRS, respectively) were assessed using penalized maximum likelihood logistic regression. All analyses were conducted using the commercial package Stata, version 16.0 (http://www.stata.com).

## 3 Results

The data comprising PD PR scores, FXTAS status, level of other neurological involvements, and demographic characteristics were compiled from the records of both American and Australian cohorts of males and females carrying CGG repeat expansions within the premutation range (*M* = 88.27, SD = 20.84). Although the data was available in 250 carriers aged from 39 to 86 years (*M* = 63.4, SD = 9.79), the number of the available data points (except PD PRS) varied between individuals. There were 184 carriers in this sample diagnosed with FXTAS, and here we split this group into mild (*N* = 52) and severe (*N* = 132) forms of this syndrome. Amongst the remaining group of 66 non‐FXTAS carriers, 56 subject were asymptomatic (*N* = 56), and 10 were monosymptomatic, that is, manifesting isolated neurological changes reminiscent of FXTAS. Distribution of PD PR scores by these categories, ranked according to the level of neurological involvement from 0 to 4, is illustrated by boxplot (Figure 1A) and Kernel density estimation (Figure 1C). The medians of standardised PD PR scores for each respective category were: −0.483 (interquartile [IQR] range = 1.32)—for asymptomatic, 1.02 (IQR = 1.05)—for single neurological changes, 0.051 (IQR = 1.35)—for mild FXTAS, and 0.103 (IQR = 1.30)—for severe FXTAS. The results of comparison of the PD PRS medians between the above categories showed significant difference (*p* = 0.038), with higher median values in other than asymptomatic categories (boxplot), and an obvious shift towards higher PD PR scores in other than asymptomatic categories. This is especially striking, but unexpected, in the category of non‐syndromic neurological involvement, but our sample is too small to consider possible reason for this exaggeration, and none of these carriers manifested parkinsonism. However, a comparison of median PD PR scores between mild and severe FXTAS groups (2, 3) combined, and combined non‐FXTAS (0–1) groups, did not show a significant difference (*p* = 0.062).

Considering the above results, and the fact that one of the two non‐FXTAS categories (1) actually encompasses the affected individuals with isolated neurological changes, the most relevant comparison in this study was conducted using asymptomatic (0) versus the sum of all three affected (1–3) categories termed here FXTAS+. This was after confirming that the difference between PD PR median scores between these categories were nonsignificant (*p* = 0.406).

The mean age of individuals in the FXTAS+ group was 65.9 years (SD = 8.46), which was significantly higher than in the asymptomatic group (Mean = 54.7 years, SD = 9.18) (*p* < 0.0001). Also predictably, there was higher proportion of males than females (66.0% vs. 48.4%, *p* = 0.040) in the affected group.

The results of comparison are illustrated in Figure 1B,D, which shows the distribution of the PD PR scores by FXTAS+ status. The median of standardised PD PR scores for new group FXTAS+ was 0.070 (IQR = 1.42).

Both boxplot (B) and Kernel distribution (D) show the shift of the higher PD PR scores towards the affected FXTAS+ group, and the result of the two‐group test showed significant difference between the medians from the asymptomatic versus FXTAS+ groups (*p* = 0.009).

The association of PD PRS with neurological involvement in context with the other known risk factors has been explored by fitting univariate and multiple logistic regression models to the data on the above categories (Table 1). The results of univariate analysis showed that FXTAS+ status is significantly associated with age at testing (*p* < 0.001), which is predictable in late‐onset conditions, sex (*p* = 0.026), which is predictable considering the protective effect of the second normal X in this sex‐linked disorder and with PD PRS (*p* = 0.021). Notably, the contribution of PD PRS remained significant even after adjusting for age and sex (*p* = 0.044) in multivariate analysis.

**TABLE 1:** Association between FXTAS+ and PD PRS, CGG repeats, and demographic characteristics using logistic regression.

	Univariate			Multivariate		
	OR	*p*	95% CI	aOR	*p*	95% CI
Only PD PRS						
Age (year)	1.149	< 0.001	1.10–1.20	1.144	< 0.001	1.10–1.19
Sex (male)	2.012	0.026	1.09–3.73	1.309	0.474	0.63–2.73
PD PRS	1.449	0.021	1.06–1.99	1.433	0.044	1.01–2.04
PD PRS and CGG repeats						
Age (year)	1.150	< 0.001	1.10–1.20	1.144	< 0.001	1.09–1.20
Sex (male)	2.290	0.011	1.21–4.33	1.671	0.212	0.75–3.74
CGG repeats	2.382	< 0.001	1.59–3.58	2.436	< 0.001	1.54–3.84
PD PRS	1.397	0.041	1.01–1.93	1.414	0.083	0.96–2.09

*Note:* Without CGG *N* = 250; With CGG *N* = 241. PD PRS and CGG repeats were standardized to have mean zero and standard deviation of 1 (*z*‐score).

Abbreviations: aOR = adjusted odds ratio; CI = confidence interval; OR = odds ratio.

**FIGURE 2:**
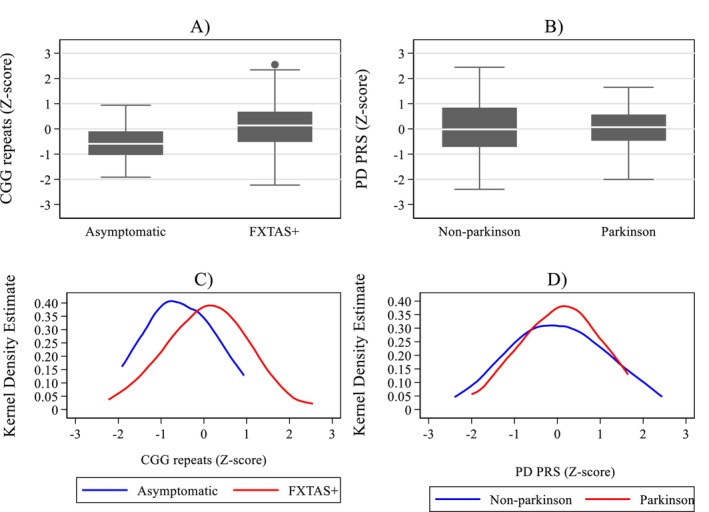
(A, C) Distributions of the CGG repeat expansion size (z‐scores) for two categories of neurological Involvement: Asymptomatic versus all affected (FXTAS+) illustrated by boxplots (A) and Kernel density estimation plots (C). (B, D) Distributions of the PD PRS (*z*‐scores) for two categories of parkinsonian status: No parkinsonism (0) versus parkinsonism (1) illustrated by boxplots (B) and Kernel density estimation plots (D).

The size of CGG expansion is a causative factor of FXTAS or any relevant neurological involvement within this spectrum (FXTAS+). The data on CGG expansion was available to us in a subsample of 241 carriers. The boxplot (Figure 2A) and Kernel Density Estimate (Figure 2C) distributions of CGG repeat size within the premutation range in asymptomatic versus all‐affected (FXTAS+) group (Figure 2B) illustrate notable effect of this size on the risk of neurological involvement in premutation carriers. More specific results of including this major CGG risk factor in the regression model (detailed in the lower section of Table 1) showed significant associations of FXTAS+ with age, sex, CGG repeats, and PD PRS in the univariate analyses (all *p* < 0.02). Predictably, in the multiple analysis, the relationship of FXTAS+ remained significant with age and CGG repeats (*p* < 0.001 for both), but it becomes weaker for PD PRS (*p* = 0.083); it would remain within significance range, however, if one‐sided test were applied, considering that we are cognisant of a direction of this score's effect on the clinical status of the PM carriers.

It is critical for further interpretation of the above result to confirm that there is no interaction between these two major predictors. Indeed, the interaction term between CGG repeats and PD PRS explored in the model was not significant (*p* = 0.550).

Since the elements of parkinsonism are seen in a proportion of the premutation carriers, especially in individuals affected with FXTAS, it was of interest to explore if there is any association between the risk of occurrence of these features and PD PRS variation. There were 210 individuals with the information on PD status represented by binary 0 or 1 scores, for an absence or presence of parkinsonism, respectively. Amongst 168 symptomatic (FXTAS+) carriers, 42 individuals scored 1, and 126 scored 0. Predictably, the association between PD status and FXTAS+ status was significant (OR = 9.01; *p* = 0.010; 95% CI = 1.70, 47.7); however, there was no relationship between PD categories and PD PRS (OR = 1.04; *p* = 0.817; 95% CI = 0.74, 1.46). Negligible effect of PD PRS on the presence of parkinsonism as observed in our sample is illustrated by boxplots and Kernel distributions in Figure 2B,D, respectively, where the difference in the medians for PD PRS (*z*‐scores) between the groups with the presence (1) or absence (0) of parkinsonism was insignificant (*p* = 0.674).

## 4 Discussion

Since the first description of FXTAS in a sample of male premutation carriers (Hagerman et al. 2001), there is still no explanation as to why neurological involvement affects only a proportion of individuals carrying these alleles, often regardless of the size of CGG expansion within the premutation range. Additionally, it remains unexplained why a proportion of individuals affected with FXTAS manifest parkinsonian features. A subsequent availability of whole genome screening techniques provides an opportunity of identifying genetic factor(s) which might account for such a modification of neurological profile. In our earlier study (Loesch, Tassone, et al. 2021), we applied a nonparametric linkage analysis, where Parkinson's disease polygenic risk scores (PD PRS) based on 107 known risk variants were calculated for the six siblings carrying premutation/grey zone alleles who consistently manifested kinetic tremor phenotypes. Suggestive evidence of linkage to a broad region of the short arm of chromosome 10 was obtained, with the median PD PRS for the sibship falling within the top 30% of a sample of over 500,000 UK and Australian controls.

In this study, we explored a possible association of PD PRS with clinical (neurological) involvement in a sample of unrelated male and female carriers of the *FMR1* premutation alleles, using the data from the affected and non‐affected individuals. We have obtained first evidence for the relationship between PD PRS and the risk of FXTAS‐like neurological involvement in premutation carriers, independent on the causal association between this involvement and CGG repeat size.

If our results can be confirmed in a much larger sample in further studies, the question remains as to the nature of the contribution of the PD PRS genetic modifier in elevating the risk of neural involvement in a separate neurodegenerative condition FXTAS. The whole of our results suggest that the answer may be sought by considering convergence of certain aspects of underlying cellular pathological mechanisms of these two disorders. In PD, mitochondrial dysfunction leading to lysosomal impairment, which can be linked to lysosome‐associated genetic risk factors for PD, has been well established (reviewed in: Mächtel et al. (2023)); in FXTAS, a number of studies provided strong evidence for the role of mitochondrial dysfunction in the pathogenesis of this disorder (reviewed in: Tassone et al. (2023)), whereby build‐up of autophagy by‐products may lead to neuronal changes regardless specific neurodegenerative condition. The intranuclear inclusions in neurons and astrocytes throughout the brain and spinal cord, exacerbating cellular dysregulation, resemble the aggregates which are the neuropathological hallmarks of PD, indicating the role of lysosomal storage disorder in the neurodegenerative process in these disorders. At the clinical level, some cases of PD bear striking resemblance to FXTAS (Rueda, Ballesteros, and Tejada 2009); and conversely, approximately 29%–60% of FXTAS patients were misdiagnosed as parkinsonism (Juncos et al. 2011; Niu et al. 2014; Salcedo‐Arellano et al. 2020). It is therefore reasonable to conclude that our results, though based on a small sample, are suggestive of modifying effect of polygenic loci, which are primarily linked to the risk of PD. These data further suggest that the action of these loci may contribute to the severity of mitochondrial dysfunction primarily caused by the *FMR1*‐CGG small expansions, and thus increasing the risk for diverse neural involvement as seen in premutation carriers. The present results have clearly shown that this contribution is, predictably, much weaker than the causative pathogenic effect of the CGG expansion size, and therefore a much larger sample is required to confirm and further specify the nature of a complex interplay between lysosomal neurodegenerative disorders in both these conditions.

Our hypothesis claiming that the PD PRS contribution in neurological manifestations is non‐specific has been supported by our results showing no difference in the distributions of these scores between the groups of premutation carriers showing the presence or absence of parkinsonian features. There was no statistical evidence, in our data, for a contribution of PD polygenic risk factor to the risk of occurrence of parkinsonism in the carriers affected with the constellation of FXTAS‐like changes. However, we are aware of possible inaccuracy of our scores for the presence or absence of parkinsonism, since the criteria for this status differed somewhat between the two cohorts, and there was an element of subjectivity in individual assessments. Another purpose of including this (negative) result in our presentation has essentially been to point out the importance of addressing this particular aspect of alleged PD PRS effect in neurological involvement in premutation carriers in future studies, based on larger cohorts and an accurate measure of parkinsonian phenotypes in these carriers.

Finally, our results provide indirect support to the notion of broad FXTAS spectrum introduced in our earlier publication (Loesch and Hagerman 2012) that is, outside of FXTAS diagnosed following the established criteria (Hall et al. 2014). The fact that the median for PD PR score becomes significantly higher for the group comprising various forms of neurological involvement versus asymptomatic category, instead of the “diagnosable” FXTAS only category compared with the “non‐FXTAS” category suggests that the syndromic and non‐syndromic forms may reflect a continuation of neurodegenerative process linked to the *FMR1* premutation allele, with the age‐ and individual‐dependent diverse final outcomes.

Although our study is the first attempt to explore a possibility that the PD polygenic risk factor is a genetic modifier of a risk of FXTAS‐like neurological involvement in a sample of unrelated carriers of premutation alleles, it is not free of certain limitations. A major factor is a relatively small sample, implying limited power and scope of statistical analysis, especially acknowledging generally small effect of genetic modifiers against the background of a primary genetic cause. Another source of bias may be related to the fact that the data have been obtained from two different sources, implying some inaccuracy of clinical classification. This may also affect ascertainment and pre‐selection of cases or each study.

In conclusion, our results based on the genome‐wide study and assessment of a clinical status are suggestive of modifying effects of genetic risk loci linked to PD, on the risk of neurological involvement in *FMR1*‐CGG small expansion carriers, resulting in a wide spectrum and severity of FXTAS‐like symptoms. Future studies based on larger sample, especially of the monosymptomatic carriers, are required to support these findings, and to explore the nature of the postulated cumulative effect of both: primary genetic cause, and PD‐ polygenes, on pathological process underlying neurological involvement in these carriers. Moreover, it is recommended that mitochondria‐specific/lysosomal burden polygenic risk scores are explored amongst other potential genetic modifiers of the broad FXTAS spectrum.

## Author Contributions

**Danuta Z. Loesch:** conceptualization; methodology; investigation; data curation; writing original draft; funding acquisition; resources; project administration; supervision. **Freddy Chafota:** investigation; formal analysis; resources; software; writing – review and editing. **Minh Q. Bui:** formal analysis; data curation; software; visualization; writing – review and editing. **Elsdon Storey:** conceptualization; methodology; investigation; resources; funding acquisition; writing – review and editing. **Anna Atkinson:** investigation; data curation; project administration; writing – review and editing. **Nicholas G. Martin:** investigation; resources; software; funding acquisition; supervision; writing – review and editing. **Scott D. Gordon:** investigation; resources; writing – review and editing. **Miguel E. Rentería:** investigation; software; writing – review and editing. **Randi J. Hagerman:** project administration; investigation; resources; funding acquisition; supervision ; writing – review and editing. **Flora Tassone:** project administration; investigation; resources; funding acquisition; supervision; writing – review and editing.

## Ethics Statement

This study was approved by the La Trobe University Human Research Ethics Committee (HEC01‐85 and HEC15‐058) and by The University of California Health IRB committee. Participants provided their informed, written consent to participate.

## Conflicts of Interest

The authors declare no conflicts of interest.

## Data Availability

The data that support the findings of this study are available from the corresponding and senior authors, D.Z.L. and F.T., upon reasonable request.
